# Total and cause-specific standardized mortality ratios in patients with schizophrenia and/or substance use disorder

**DOI:** 10.1371/journal.pone.0202028

**Published:** 2018-08-23

**Authors:** Ina H. Heiberg, Bjarne K. Jacobsen, Ragnar Nesvåg, Jørgen G. Bramness, Ted Reichborn-Kjennerud, Øyvind Næss, Eivind Ystrom, Christina M. Hultman, Anne Høye

**Affiliations:** 1 Center for Clinical Documentation and Evaluation (SKDE), Tromsø, Norway; 2 Department of Community Medicine, UiT—The Arctic University of Norway, Tromsø, Norway; 3 Nydalen DPS, Oslo University Hospital, Oslo, Norway; 4 Norwegian National Advisory Unit on Concurrent Substance Abuse and Mental Health Disorders, Innlandet Hospital Trust, Hamar, Norway; 5 Department of Clinical Medicine, Faculty of Health Sciences, UiT—The Arctic University of Norway, Tromsø, Norway; 6 Department of Mental Disorders, Norwegian Institute of Public Health, Oslo, Norway; 7 Institute of Clinical Medicine, University of Oslo, Oslo, Norway; 8 Institute of Health and Society, University of Oslo, Oslo, Norway; 9 Department of Psychology, University of Oslo, Oslo, Norway; 10 PharmacoEpidemiology and Drug Safety Research Group, School of Pharmacy, University of Oslo, Oslo, Norway; 11 Department of Medical Epidemiology and Biostatistics, Karolinska Institutet, Stockholm, Sweden; 12 Division of Mental Health and Substance Abuse, University Hospital of North Norway, Tromsø, Norway; Institute of Psychiatry, UNITED KINGDOM

## Abstract

Individuals with schizophrenia or substance use disorder have a substantially increased mortality compared to the general population. Despite a high and probably increasing prevalence of comorbid substance use disorder in people with schizophrenia, the mortality in the comorbid group has been less studied and with contrasting results. We performed a nationwide open cohort study from 2009 to 2015, including all Norwegians aged 20–79 with schizophrenia and/or substance use disorder registered in any specialized health care setting in Norway, a total of 125,744 individuals. There were 12,318 deaths in the cohort, and total, sex-, age- and cause-specific standardized mortality ratios (SMRs) were calculated, comparing the number of deaths in patients with schizophrenia, schizophrenia only, substance use disorder only or a co-occurring diagnosis of schizophrenia and substance use disorder to the number expected if the patients had the age-, sex- and calendar-year specific death rates of the general population. The SMRs were 4.9 (95% CI 4.7–5.1) for all schizophrenia patients, 4.4 (95% CI 4.2–4.6) in patients with schizophrenia without substance use disorder, 6.6 (95% CI 6.5–6.8) in patients with substance use disorder only, and 7.4 (95% CI 7.0–8.2) in patients with both schizophrenia and substance use disorder. The SMRs were elevated in both genders, in all age groups and for all considered causes of death, and most so in the youngest. Approximately 27% of the excess mortality in all patients with schizophrenia was due to the raised mortality in the subgroup with comorbid SUD. The increased mortality in patients with schizophrenia and/or substance use disorder corresponded to more than 10,000 premature deaths, which constituted 84% of all deaths in the cohort. The persistent mortality gap highlights the importance of securing systematic screening and proper access to somatic health care, and a more effective prevention of premature death from external causes in this group.

## Introduction

Individuals with schizophrenia have a two- to three-fold increased mortality compared to the general population [1–3] and a 10-20-year reduction in average life span [2, 4–6]. Individuals with substance use disorder (SUD) have an even higher risk of premature death, ranging from a four-fold increased mortality among persons with alcohol use disorder (AUD) [7–10] to a four to-15-fold increased mortality among opioid users [11–13]. A concurrent diagnosis of schizophrenia spectrum disorder (SCZ) and SUD (henceforth referred to as SCZ+SUD) is associated with a variety of detrimental outcomes, among these increased somatic morbidity [14–19], increased risk of fatal overdoses, violent behavior [20–23] and victimization [24, 25]. Despite these vulnerabilities and a high [26, 27], and probably increasing [28], prevalence of comorbid SUD in SCZ patients, the mortality in the comorbid group has been less studied and with contrasting results. Studies of SCZ individuals with co-morbid SUD have found both increased [29–34] and decreased [35] all-cause mortality, increased suicide mortality [36, 37], but no difference in cardiovascular mortality [38] compared to SCZ individuals without SUD. Studies of SUD individuals report both higher [39, 40], similar [12, 13, 40–43], and lower [44] mortality in individuals also diagnosed with a psychotic disorder, compared to individuals with SUD-only, depending on type of SUD and gender [40]. Only a few studies with complete national coverage have investigated all cause [30, 33, 34, 40] or cause-specific mortality [33, 34] in patients with SCZ-only, SUD-only or co-morbid SCZ and SUD, and neither of these reported results for different age groups or mortality from unnatural causes of death.

The aims of the present study were to (i) investigate standardized mortality ratios for all cause mortality (SMRs) in patients with SCZ, SCZ-only, SUD-only and SCZ+SUD, diagnosed in Norwegian psychiatric or somatic specialist health care, (ii) describe how much of the excess mortality that could be attributed to a concurrent diagnosis of SCZ and SUD, and (iii) investigate age-, sex-, and cause-specific SMRs in patients with SCZ-only, SUD-only or SCZ+SUD. We hypothesized that mortality in patients with SCZ and/or SUD would be increased compared to the general population for all main causes of death, and that patients with a concurrent diagnosis of SCZ and SUD would have particularly high SMRs.

## Materials and methods

### Study population

The study population included all in- and outpatients diagnosed with SCZ and/or SUD in the Norwegian specialized health care system during 2009–2015, who were 20–79 years old when they had their first consultation in the time bracket. Patients with SCZ and/or SUD were identified through the Norwegian Patient Registry (NPR), which is a mandatory national registry covering all patients receiving specialist health care (i.e. government-owned hospitals and outpatient clinics, publicly financed substance use treatment facilities and private health clinics with governmental reimbursement). Since 2009 the coverage in the NPR is almost 100% [45, 46]. The exception is private mental health clinics with governmental reimbursement, where the proportion of the episodes that were reported to the NPR increased from 76% in 2009 [47] to 97% in 2015 [48], but only 0.2% of patients in the present study were treated solely in these clinics. Patients were excluded if an invalid ID number (n = 6,178), a diagnosis of intellectual disability (ICD-10 codes F70-F79) (n = 1,632), or inconsistent data regarding age, gender or emigration date (n = 666) were recorded.

### Diagnostic categories

Diagnostic codes in the NPR follow the International Classification of Diseases and Related Health Problems, 10th Revision (ICD-10) [49]. Patients were included in the SCZ group if a diagnosis of schizophrenia-related disorders (F20-F29) was recorded in the NPR during 2009–2015. The SUD group included patients with a diagnosis of mental and behavioral disorders due to use of psychoactive substances (F10-F19, excluding tobacco (F17)), and patients with at least one somatic diagnosis strongly indicating alcohol abuse (see list of ICD-10 codes in S1 Table). Patients identified by somatic diagnoses only constituted 4.1% of the SUD group. A total of 275 patients treated in substance use treatment facilities, with a diagnosis of SCZ, but no registered diagnosis of SUD (excluding pathological gambling (F63.0)), were included in the SCZ+SUD group. In subgroup analyses, we differentiated between patients with a non-alcohol SUD (F11-F16, F18-F19, Z50.3, Z71.5 and Z72.2), and patients with AUD only. Hard drug use disorder was deemed present if the patient was diagnosed with disorders due to use of opioids (F11), cocaine (F14), other stimulants (mostly amphetamines, F15), hallucinogens (F16), volatile solvents (F18), and multiple drug use and use of other psychoactive substances (F19).

### Information on deaths

The Cause of Death Registry (CDR), which includes 98% of all deaths in Norway [50], provided data on the underlying cause of death, based on death certificates issued by physicians. The National Population Registry provided information about year of birth and date of death or emigration. Accurate linkage regarding mortality of the patients was obtained using the unique 11 digit personal ID number included in all the registries. Causes of death were coded according to ICD-10 [49] and divided into natural causes (A00-R99), unnatural causes (V01-Y98) and missing cause of death. Natural causes of death were further divided into cardiovascular (I00-I99), respiratory (J00-J99), cancer (C00-C97) and other natural causes. Unnatural causes of death were divided into poisoning (X40-X49), suicide (X60-X84 and Y87.0) and other unnatural causes.

The reference population included all residents in Norway aged 20–79 during 2009–2015. Annual number of deaths in gender-stratified five-year age groups for the reference population were obtained from the Norwegian Institute of Public Health [51], and annual population figures in the age groups 20–79 in the years 2009–2015 were obtained from Statistics Norway [52].

### Follow-up

The study cohort was followed from the admission date of the index episode (their first consultation during 2009–2015). Patients already hospitalized before January 1, 2009, were followed from this date (n = 2,633). To account for diagnostic instability in SCZ patients, we defined an extended diagnostic spectrum covering schizophrenia (F20-F29), affective disorders (F30-F39), and personality disorder (F60). If a diagnosis of SCZ was ever recorded, the index episode was defined as the first episode with a diagnosis within the extended diagnostic spectrum. Follow-up ended on December 31, 2015, on the date of emigration from Norway, on December 31 in the year of the 79^th^ birthday, or on the date of death, whichever came first.

### Statistical analysis

For comparison with the mortality of the general population of Norway, SMRs with corresponding 95% confidence intervals were calculated for patients with SCZ, SCZ-only, SUD-only, and SCZ+SUD. SMR reflects the relative mortality of the patient group compared to that of the general population and is computed as the observed number of deaths divided by the expected number of deaths. The expected number of deaths was calculated as the total number of person-years at risk in each sex-, age group- and calendar year band, multiplied by the corresponding age- (5-year age groups), sex- and calendar-year specific (2009–2015) death rate in the general population. Age was defined as attained age at the end of each calendar year, as we did not have access to exact birth dates. Person-years at risk contributed by persons who moved from one age band to the next during follow-up was assigned to the respective sex-, age group- and calendar year bands, using the “lexis” method [53]. The number of excess deaths was calculated as the difference between observed and expected deaths.

Biased SMRs may be a major problem when the prevalence of exposure in the general population is high [54]. In contrast to SCZ, unnatural deaths in the SUD subgroup constitute a large proportion of unnatural deaths in the general population, implicating risk of biased SMRs for unnatural causes of death in this subgroup. We consequently investigated the impact on SMRs for poisoning, suicide and all-cause mortality in patients with SUD (SUD-only and SCZ+SUD combined), using an unexposed comparison group, rather than the general population, for calculation of the expected number of deaths. This unexposed comparison group constituted the Norwegian population excluding subjects with recorded SUD in specialized health care and corresponding deaths from the relevant age-/gender group in the reference population. The mortality rates in this unexposed population was calculated and applied when computing the expected number of deaths.

We also investigated the impact on SMRs of the temporal ordering of SCZ and SUD diagnoses in comorbid patients, and of varying inclusion criteria in a series of pre-specified sensitivity analyses (i) excluding patients treated solely in somatic hospitals during follow-up (e.g. older individuals with presumably stable mental illness suffering from somatic health problems), (ii) excluding patients diagnosed with SCZ as a secondary diagnosis only, and (iii) including only patients diagnosed with narrow schizophrenia (F20) in the SCZ group.

The analyses were conducted using SAS statistical software, version 9.4 (SAS Institute Inc., Cary, N.C.)

### Ethics

All patient data were fully de-identified when accessed by the investigators. In Norway, studies with de-identified information from medical health registries do not require participant consent. Legal basis and exemption from professional secrecy requirements for the use of personal health data in research was granted by the regional committee for medical and health research ethics (2014/72/REK nord).

## Results

### Characteristics of the study population

A total of 125,744 individuals were included in the study during 2009–2015. They were aged 20–79 at start of follow-up. Of these, 27,859 (22.2%) were registered with a diagnosis of SCZ, 20,537 (16.3%) with SCZ-only, 97,885 (77.8%) with SUD-only, and 7,322 (5.8%) with SCZ+SUD (Table 1). Men were overrepresented in the SUD groups (66.6% in SUD-only and 70.1% in SCZ+SUD). In patients with SUD, AUD was most common in patients with SUD-only (66.4%), whereas non-alcohol SUD was most common in comorbid patients (76.5%).

**Table 1:** Characteristics of the study population. Patients aged 20–79 with schizophrenia spectrum disorder (SCZ) and/or substance use disorder (SUD).

				SCZ-only		SUD-only		SCZ+SUD	
Men									
	Patients, n (%)			10,509		65,175		5,126	
	Personyears, sum			48,776		250,350		26,873	
	Personyears, mean (SD)			4.6	(2.3)	3.8	(2.2)	5.2	(2.0)
	Age at start of follow-up, mean (SD)			43.4	(15.2)	43.5	(15.7)	35.2	(12.0)
	No. of patients with substance use disorder, n (%)								
		Alcohol use disorder		-		44,198	(67.8)	2,230	(43.5)
		Alcohol use disorder only		-		33,917	(52.0)	980	(19.1)
		Non-alcohol substance use disorder		-		31,258	(48.0)	3,984	(77.7)
			Cannabis use disorder	-		13,688	(21.0)	1,805	(35.2)
			Hard drug use disorders	-		23,073	(35.4)	3,202	(62.5)
		Non-alcohol substance use disorder only		-		20,977	(32.2)	2,734	(53.3)
		Unknown substance use disorder		-		0	(0.0)	162	(3.2)
	No. of health care episodes per person-year, median (IQR)								
		No. of admissions, mental care		0.2	(0.0–0.4)	0.0	(0.0–0.0)	0.3	(0.2–0.6)
		No. of outpatient visits, mental care		5.6	(1.1–16.5)	0.0	(0.0–2.0)	7.3	(1.9–19.0)
		No. of admissions, medical care		0.0	(0.0–0.2)	0.2	(0.0–0.4)	0.1	(0.0–0.3)
		No. of outpatient visits, medical care		0.6	(0.0–1.9)	1.5	(0.5–4.2)	0.9	(0.3–2.0)
		No. of admissions, SUD treatment		-		0.0	(0.0–0.2)	0.0	(0.0–0.1)
		No. of outpatient visits, SUD treatment		-		0.2	(0.0–3.9)	0.0	(0.0–1.8)
Women									
	Patients, n (%)			10,028		32,710		2,196	
	Personyears, sum			45,970		126,174		11,946	
	Personyears, mean (SD)			4.6	(2.3)	3.9	(2.2)	5.4	(1.9)
	Age at start of follow-up, mean (SD)			47.9	(16.1)	42.5	(16.2)	39.1	(14.0)
	No. of patients with substance use disorder, n (%)								
		Alcohol use disorder		-		20,800	(63.6)	965	(43.9)
		Alcohol use disorder only		-		15,471	(47.3)	442	(20.1)
		Non-alcohol substance use disorder		-		17,239	(52.7)	1,641	(74.7)
			Cannabis use disorder	-		4,573	(14.0)	433	(19.7)
			Hard drug use disorders	-		11,834	(36.2)	1,247	(56.8)
		Non-alcohol substance use disorder only		-		11,910	(36.4)	1,118	(50.9)
		Unknown substance use disorder		-		0	(0.0)	113	(5.1)
	No. of health care episodes per person-year, median (IQR)								
		No. of admissions, mental care		0.2	(0.0–0.4)	0.0	(0.0–0.2)	0.4	(0.2–0.7)
		No. of outpatient visits, mental care		5.8	(1.2–17.0)	0.8	(0.0–6.1)	8.4	(2.5–21.6)
		No. of admissions, medical care		0.1	(0.0–0.3)	0.2	(0.0–0.5)	0.2	(0.0–0.5)
		No. of outpatient visits, medical care		1.0	(0.2–3.1)	2.4	(0.9–6.0)	1.5	(0.6–3.3)
		No. of admissions, SUD treatment		-		0.0	(0.0–0.0)	0.0	(0.0–0.0)
		No. of outpatient visits, SUD treatment		-		0.0	(0.0–3.7)	0.0	(0.0–1.6)

Abbreviations: SCZ, schizophrenia spectrum disorder; SUD, substance use disorder; SCZ+SUD, concurrent diagnoses of schizophrenia and substance use disorder; SD, standard deviation; IQR, interquartile range.

### All-cause mortality

**Fig 1:**
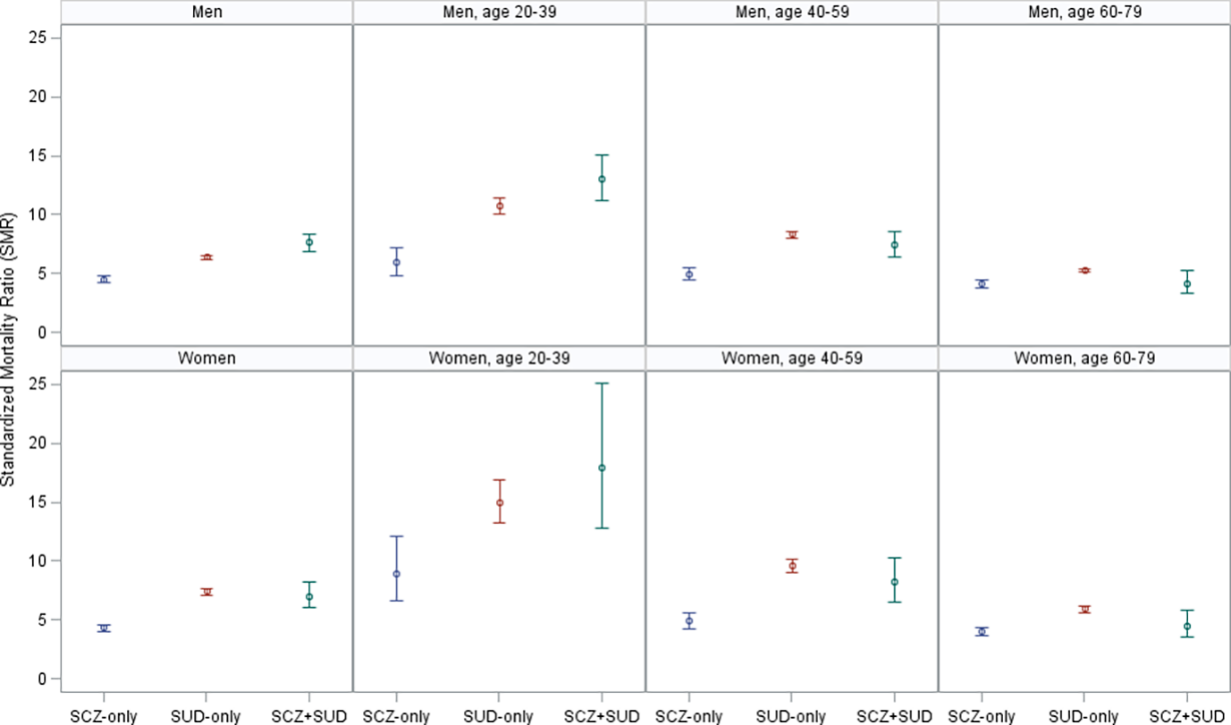
All-cause age, gender, and calendar-year standardized mortality ratios according to gender and age among patients with schizophrenia and/or substance use disorders.

Of the 125,744 individuals included, 12,318 (9.8%) died during follow-up (Table 2). Mean age at death was highest in patients with SCZ-only (62.0 years) and lowest in patients with SCZ+SUD (47.3 years), and particularly low in comorbid men (45.3 years). Comparing with the expected number of deaths based on the mortality of the general population, we found a SMR of 4.9 (95% CI 4.7–5.1) in SCZ patients (with or without SUD), and SMRs of 4.4 (95% CI 4.2–4.6), 6.6 (95% CI 6.5–6.7) and 7.4 (95% CI 6.9–8.1) in patients with SCZ-only, SUD-only, and SCZ+SUD, respectively, corresponding to 10,328 excess deaths (of which 18% were in SCZ patients). In men, the SMRs for patients with SCZ (with or without SUD), SCZ-only, SUD-only, and SCZ+SUD were 5.1 (95% CI 4.8–5.4), 4.5 (95% CI 4.2–4.8), 6.4 (95% CI 6.2–6.5) and 7.6 (95% CI 6.9–8.4), respectively. In women, the corresponding figures were 4.6 (95% CI 4.3–4.9), 4.3 (95% CI 4.0–4.6), 7.4 (95% CI 7.1–7.6) and 7.0 (95% CI 6.0–8.2). All-cause mortality was elevated for both genders and in all age groups (Fig 1), but the SMRs were consistently higher in the youngest age groups. Women aged 20–39 years with SUD had particularly high SMRs; 15 in the SUD-only group and 18 in the SCZ+SUD group.

**Table 2:** Number of deaths (overall and in different age groups) and mean age of death, according to sex. Patients aged 20–79 with schizophrenia spectrum disorder (SCZ) and/or substance use disorder (SUD).

			SCZ-only		SUD-only		SCZ+SUD	
Men								
	Deaths, n		972		7,334		423	
		Age at death 20–39, n (%)	90	(9.3)	847	(11.5)	170	(40.2)
		Age at death 40–59, n (%)	295	(30.3)	2,445	(33.3)	171	(40.4)
		Age at death 60–79, n (%)	587	(60.4)	4,042	(55.1)	82	(19.4)
	Age at death, mean (SD)		60.3	(13.6)	58.6	(13.7)	45.3	(14.2)
Women								
	Deaths, n		832		2,592		165	
		Age at death 20–39, n (%)	42	(5.0)	256	(9.9)	34	(20.6)
		Age at death 40–59, n (%)	198	(23.8)	928	(35.8)	75	(45.5)
		Age at death 60–79, n (%)	592	(71.2)	1,408	(54.3)	56	(33.9)
	Age at death, mean (SD)		64.0	(12.3)	59.0	(13.6)	52.5	(14.2)

Abbreviations: SCZ, schizophrenia spectrum disorder; SUD, substance use disorder; SCZ+SUD, concurrent diagnoses of schizophrenia and substance use disorder; SD, standard deviation.

Sensitivity analyses (i) excluding patients treated solely in somatic hospitals during follow-up, or (ii) patients with a secondary diagnosis of SCZ only indicated decreased SMRs for SCZ-only (both models) and SUD-only (model ii), whereas application of a narrow definition of SCZ (F20) (model iii) resulted in moderately increased SMRs for SCZ-only (S2 Table).

### Impact of a concurrent diagnosis of SCZ and SUD

Overall, comorbid patients had higher all-cause SMRs than patients with SCZ-only or SUD-only. However, when stratified according to sex and type of SUD (Table 3), SMRs were similar in SUD patients with or without comorbid SCZ, except in comorbid men with a non-alcohol SUD who had higher SMR than men with non-alcohol SUD-only. Comorbid patients aged 20–39, 40–59 and 60–69 had 107%, 57% and 6% higher numbers of deaths compared to what could be expected if the same age-specific SMRs as patients with SCZ-only had applied, whereas application of age-specific SMRs for SUD-only in the comorbid had smaller impact on hypothetical numbers of death. Thus, the major reason for the high SMRs in comorbid patients was the SUD component. In this total Norwegian patient population, approximately 27% of the excess number of deaths in all patients with SCZ was due to the raised mortality in the subgroup with comorbid SUD. This effect was strongest in men and in the youngest.

**Table 3:** All-cause age, gender, and calendar-year standardized mortality ratios among patients aged 20–79 with substance use disorders only (SUD-only) or concurrent SUD and schizophrenia related disorders (SCZ+SUD), according to type of SUD.

		SUD-only			SCZ+SUD		
		Obs	SMR^a^	(95% CI)	Obs	SMR^a^	(95% CI)
Men		7,334	6.4	(6.2–6.5)	423	7.6	(6.9–8.3)
	AUD-only	5,109	5.9	(5.7–6.0)	120	5.4	(4.5–6.4)
	Non-alcohol SUD	2,225	8.0	(7.7–8.3)	295	9.4	(8.4–10.5)
	Unknown SUD	0			8	4.0	(2.0–8.0)
Women		2,592	7.4	(7.1–7.7)	165	7.0	(6.0–8.2)
	AUD-only	1,520	7.0	(6.6–7.3)	47	6.4	(4.8–8.5)
	Non-alcohol SUD	1,072	8.0	(7.5–8.5)	111	7.4	(6.1–8.9)
	Unknown SUD	0			7	6.9	(3.3–14.4)

Abbreviations: SUD, substance use disorder; SCZ+SUD, concurrent diagnoses of schizophrenia and substance use disorder; Obs, observed deaths; SMR, standardized mortality ratio; 95% CI, 95% confidence interval; AUD, Alcohol Use Disorder.

^a^ The standard population used for the sex- and calendar year specific age standardization was the annual population of Norway aged 20–79 in the years 2009–2015.

Supplementary analyses examining the temporal ordering of SCZ and SUD diagnoses in comorbid patients showed that SCZ and SUD were mostly co-occurring: 39% had both diagnoses within three months, 55% within one year and 70% within two years. One in four comorbid patients had a SUD diagnosis more than three months prior to the SCZ diagnosis, whereas 36% had a SCZ diagnosis more than three months prior to the SUD diagnosis. The SMRs tended to be higher when the SUD diagnosis preceded the SCZ diagnosis, and were highest when the first diagnosis of SCZ and SUD during follow-up were recorded within a short time interval (S3 Table).

### Cause specific mortality

We found elevated mortality from all considered causes of death in all subgroups (Table 4). In both men and women, the highest SMRs were observed for poisoning, suicides and respiratory diseases. Women had higher SMRs for unnatural death than men in all age groups, and higher SMRs for suicides. SMRs for natural causes of death did not differ much with age neither for men nor for women. For unnatural causes of death, SMRs in people with SCZ-only were stable across age groups, except in women aged 20–39, who had elevated SMRs compared to other age groups. In patients with SUD, SMRs in the age group 20–59 were elevated compared to age 60–79, except in comorbid women who had a 30-fold increased SMR across all age groups. Overall, 73% of excess number of deaths were from natural causes. In SCZ-only and SUD-only, natural causes accounted for 82% and 73% of all deaths, respectively, with cancer and cardiovascular disease being the most common causes. In the SCZ+SUD group, unnatural causes accounted for 54% of all deaths. Women with SUD-only had higher SMRs than men with SUD-only for nearly all considered causes of death. Women with SUD (with or without SCZ) had particularly high SMRs (approximately 45) for poisoning.

**Table 4:** Cause-specific age, gender, and calendar-year standardized mortality ratios among men and women aged 20–79 with schizophrenia-related disorders (SCZ) and/or substance use disorders (SUD).

				SCZ-only			SUD-only			SCZ+SUD		
					Obs	SMR^a^	95% CI	Obs	SMR^a^	95% CI	Obs	SMR^a^	95% CI
Men												
	Natural causes			775	4.2	(3.9–4.5)	5,293	5.4	(5.3–5.6)	182	4.6	(4.0–5.3)
			Age 20–39	17	3.3	(2.1–5.3)	141	5.5	(4.6–6.4)	24	5.7	(3.8–8.5)
			Age 40–59	211	4.7	(4.1–5.3)	1,555	6.9	(6.6–7.2)	86	5.1	(4.1–6.3)
			Age 60–79	547	4.1	(3.8–4.5)	3,597	5.0	(4.8–5.2)	72	3.9	(3.1–5.0)
		Subtype										
			Cardiovascular	200	3.9	(3.4–4.5)	1,252	4.7	(4.4–4.9)	35	3.3	(2.4–4.6)
			Respiratory	123	8.5	(7.1–10.1)	617	8.0	(7.4–8.7)	30	13.1	(9.1–18.7)
			Cancer	197	2.6	(2.3–3.0)	1,180	2.9	(2.7–3.1)	30	1.9	(1.3–2.7)
			Other	255	6.1	(5.4–6.9)	2,244	10.2	(9.8–10.7)	87	8.3	(6.7–10.3)
	Unnatural causes				186	6.9	(5.9–7.9)	1,837	13.3	(12.7–13.9)	228	16.3	(14.3–18.5)
			Age 20–39	72	7.8	(6.2–9.8)	684	14.1	(13.1–15.2)	140	17.4	(14.8–20.6)
			Age 40–59	76	6.4	(5.1–8.0)	807	14.2	(13.3–15.3)	81	16.0	(12.9–19.9)
			Age 60–79	38	6.4	(4.6–8.7)	346	10.4	(9.4–11.6)	7	7.6	(3.6–16.0)
		Subtype										
			Poisoning	30	4.6	(3.2–6.5)	903	27.5	(25.8–29.3)	115	29.0	(24.2–34.9)
			Suicide	115	11.6	(9.7–13.9)	499	9.9	(9.0–10.8)	87	16.0	(13.0–19.8)
			Other	41	3.8	(2.8–5.2)	435	7.9	(7.2–8.7)	26	5.6	(3.8–8.3)
	Missing			11	1.6	(0.9–2.8)	204	5.4	(4.7–6.2)	13	5.5	(3.2–9.5)
Women												
	Natural causes			703	3.9	(3.6–4.2)	1,921	6.0	(5.8–6.3)	90	4.4	(3.6–5.4)
			Age 20–39	8	3.0	(1.5–6.0)	46	5.2	(3.9–7.0)	8	7.9	(4.0–15.8)
			Age 40–59	146	4.2	(3.6–4.9)	608	7.4	(6.8–8.0)	40	5.2	(3.8–7.0)
			Age 60–79	549	3.9	(3.6–4.2)	1,267	5.6	(5.3–5.9)	42	3.6	(2.6–4.8)
		Subtype										
			Cardiovascular	150	4.3	(3.7–5.1)	306	5.4	(4.8–6.1)	17	5.1	(3.2–8.2)
			Respiratory	126	7.1	(6.0–8.4)	292	10.0	(8.9–11.2)	22	13.4	(8.9–20.4)
			Cancer	239	2.7	(2.4–3.1)	451	2.8	(2.5–3.0)	19	1.7	(1.1–2.7)
			Other	188	4.7	(4.1–5.5)	872	12.6	(11.8–13.4)	32	7.2	(5.1–10.1)
	Unnatural causes			113	9.9	(8.2–11.9)	629	23.2	(21.4–25.0)	74	30.9	(24.6–38.9)
			Age 20–39	29	15.7	(10.9–22.6)	201	26.7	(23.2–30.6)	26	32.4	(22.1–47.7)
			Age 40–59	47	9.7	(7.3–12.9)	305	25.8	(23.1–28.9)	35	29.9	(21.5–41.7)
			Age 60–79	37	7.8	(5.7–10.8)	123	15.8	(13.2–18.8)	13	30.9	(18.0–53.3)
		Subtype										
			Poisoning	11	5.1	(2.8–9.2)	263	44.7	(39.6–50.4)	25	43.5	(29.4–64.4)
			Suicide	77	19.0	(15.2–23.8)	248	22.7	(20.0–25.7)	38	36.7	(26.7–50.4)
			Other	25	4.8	(3.2–7.1)	118	11.4	(9.5–13.7)	11	14.1	(7.8–25.4)
	Missing			16	5.0	(3.1–8.2)	42	6.7	(5.0–9.1)	1	nr	nr

Abbreviations: Obs, observed deaths; SMR, standardized mortality ratio; 95% CI, 95% confidence interval; nr, Not reportable because of small samples (no. of deaths < 5) within the data category.

^a^ The standard population used for the sex- and calendar year specific age standardization was the annual population of Norway aged 20–79 in the years 2009–2015.

Deaths due to poisoning and suicide in SUD patients in our sample constituted a large proportion of all deaths from these causes in Norway in the years 2009–2015 (52% and 22%, respectively). In a supplementary analysis we assessed the bias in SMRs for poisoning and suicide for patients with SUD (SUD-only and SCZ+SUD combined) using an unexposed comparison group (e.g. no diagnosis of SUD in specialized health care), rather than the general population, to calculate expected deaths. This resulted in a three-fold increase in SMR for poisoning in men and a doubled SMR for poisoning in women (from 27.7 to 82.9 in men, and from 44.6 to 98.5 in women), whereas SMRs for suicides increased from 10.5 to 13.7 in men and from 23.9 to 33.8 in women. The SMRs for all-cause mortality were also affected (increased from 6.4 to 7.3 in men, and from 7.3 to 7.9 in women).

## Discussion

Our study shows that patients in specialized health care with SCZ, SCZ-only or SUD (with or without SCZ) had a five-, four- and seven-fold increased total mortality, respectively, compared to the general population. Mortality was elevated for both genders, in all age groups and for all considered causes of death, with the highest SMRs observed for poisoning, suicides and respiratory diseases. Very high SMRs for poisoning were noted in women with SUD (with or without SCZ). The highest SMRs were found in the age group 20–39, mainly due to unnatural causes of death.

The excess mortality corresponded to more than 10 000 premature deaths (84% of all deaths in the cohort). About 27% of the excess deaths in SCZ patients could be attributed to SUD (or factors associated with SUD). The nearly five-fold increased mortality in SCZ (with and without SUD) was substantially higher than reported in meta-analyses [1, 55], and also higher than reported in a recent Nordic study [56]. The omission of patients in primary care, and the relatively short follow-up, may have led to a selection of more severe cases, oversampling patients with many health care episodes and patients with increased suicide risk. Inclusion of older individuals suffering from somatic health problems, such as cardiovascular and respiratory diseases, is another explanation of the elevated SMR in SCZ patients, as shown in the sensitivity analysis. Differences in case finding criteria might also have contributed, also shown in the sensitivity analyses, whereas survivor bias resulting from inclusion of prevalent cases would have the opposite effect. Also, catchment of SCZ might be higher in Norway compared to countries without universal health care. Another contributing explanation might be the rising mortality gap in schizophrenia patients compared to the general population reported in recent studies [1, 57, 58], partly explained by decreased mortality from cardiovascular disease in the general population [59, 60].

The increased all cause and suicide mortality, as well as the similar cardiovascular mortality in comorbid patients, is in accordance with some previous studies [29–34, 38], but not in line with a study reporting decreased mortality in persons with co-occurring psychosis and cannabis use/abuse, compared to psychosis only [35]. In the present study, mortality from cannabis use disorder was not studied separately, due to few deaths and suspicion of selective recording of less severe cases in comorbid patients.

Overall, a co-occurring diagnosis of SCZ also conferred increased SMRs in SUD patients. However, when stratified according to type of SUD, we found similar SMRs for non-alcohol SUD patients with and without SCZ, in accordance with other studies [12, 13, 41, 42], and similar SMRs in AUD patients with and without SCZ, in contrast to a 50-year follow-up study of AUD persons [39]. Differences in length of follow-up may be one explanation for the latter finding. Furthermore, when stratified according to gender, we found increased SMR in comorbid men, but not in comorbid women, in accordance with a study conducted in inpatients [40]. Thus, in our study, the increased SMRs in comorbid patients, both genders combined, was explained by the high proportion of young comorbid men with a non-alcohol SUD. The eight-to-nine-fold increased SMR in patients with non-alcohol SUD with and without SCZ, is similar to [61] or higher than [12, 31] reported elsewhere. Norway has one of the highest rates of injecting drug users among hard drug users [62], which may have contributed to this finding.

The very high all cause SMRs found among young patients were similar to [63] or higher than [31, 64] reported earlier. Previous studies have found excess mortality to be highest the year following a first diagnosis of severe mental illness, due to increased mortality from unnatural causes of death [2, 65]. Besides low expected death rates in the youngest in the general population, the inverse relationship between the SMR and age may thus partly be explained by the inclusion of presumably more incident cases in the youngest and more prevalent cases among the oldest in our study, and may have been amplified by survivor bias, treatment-compliant patients in our sample and lower suicide rates with increasing age in SCZ patients [66]. Comorbid women did not, however, experience a decrease in SMR with increasing age, but had very high mortality across all age groups. We found very high SMRs for poisoning and suicide in women with SUD, which confirms earlier findings [60, 67], and high SMRs for respiratory diseases, probably associated with increased smoking prevalence in individuals with severe mental disorders [68]. The increased mortality due to suicide in patients with SCZ was more pronounced than reported in a systematic review [1], but of similar magnitude as reported in a sample with comparable age span [37].

The excess mortality in patients with severe mental illness has been ascribed to a wide range of factors, both at the individual level, health system level and at the societal level [69]. Explanations include lifestyle factors [38, 70] (such as smoking, substance use, physical inactivity and unhealthy diet), poorer socioeconomic conditions [71], suboptimal use of or access to medical care [72], and metabolic side effects of high dosages of antipsychotic medication [73], as well as higher levels of suicides, poisoning, violent behavior [20–23] and victimization [24, 25]. It is thus a huge challenge to address the complex needs of patients with severe mental illness in combination with SUD, both with regard to general improvement of outcome as well as prevention of premature mortality. Despite the well known high association between SUD and SCZ there is no evidence supporting one specific treatment strategy for patients with dual diagnoses [69, 74, 75]. Likewise, there is little evidence for successful interventions addressing somatic risk factors in patients with severe mental disorders [70, 76], although evidence-based interventions for smoking cessation and weight reduction in persons with severe mental illness exist [69]. The universal health care system in Norway, designed to prevent socioeconomic differences in health care, has unfortunately not been able to address these issues. Generally, a population approach for prevention is considered the key avenue for non-communicable diseases, e.g. cardiovascular diseases and cancer [77]. The patients here studied clearly constitute a high-risk group, but there is limited knowledge of effective intervention and treatment strategies to use with these vulnerable patients [4]. An approach that includes both individual focused and system focused interventions is probably needed, involving both primary and integrated specialist health care. Further research is needed to pinpoint effective system focused interventions specifically aiming at reaching the high-risk subgroup with mental illness. The extremely high mortality risk in young patients highlights the need for such efforts to be implemented in the younger age groups.

### Strengths and limitations

The major strength of this study is the use of unselected and complete nationwide registry data of recent date, covering all specialist health care settings, and with no bias associated with selective self-reporting. The inclusion of outpatients and patients treated solely in somatic hospitals also enabled more generalizable risk estimates. The CDR is found to provide high quality data on the underlying cause of death [78]. Also, the validity of a schizophrenia diagnosis in hospital case registries is good when compared to diagnoses based on structured research interviews [79, 80]. Furthermore, in Norway, treatment of psychosis is mainly a specialist task, with free admission to hospital treatment and a maximum annual cost of 2205 NOK (approximately 235 €) for outpatient treatment, implying high coverage for SCZ in the NPR.

The relatively short follow-up is a limitation, which also precluded analysis of secular trends in SMRs. Another important limitation is the lack of control for unmeasured individual risk factors associated with mortality. However, studies adjusting for such factors [30, 38, 81] still revealed an increased mortality after adjustment. The SMRs in the current study may be prone both to under- and overestimation. We found underestimated SMRs for unnatural causes of death in SUD patients when the general population, rather than an unexposed population, was used as reference. Survivor bias in comorbid patients may also have resulted in underestimation, as patients would have to live long enough to receive both diagnoses. On the other hand, underreporting of less severe SUD in specialized care may have led to overestimated SMRs in all groups, although a higher detection rate of less severe SUD in comorbid patients (Berkson’s bias) may be suspected. SCZ and SUD were identified independently of time, ignoring the possibility that the two diagnoses were not co-occurring. However, supplementary analyses showed that a majority of comorbid patients had both diagnosed within a year. We were not able to differentiate between patients with a primary psychotic disorder that co-occurs with SUD and patients with substance-induced psychosis, although we found indications that patients with a SUD diagnosis preceding SCZ had worse outcomes. We found a 26% prevalence of SUD in SCZ patients during a mean follow-up of four years, which is similar to a previous Norwegian study [27] SUD diagnoses in the NPR have not been subject to formal validity checks [80], but a Norwegian validation study found hospital diagnosis of SUD to be fairly valid, although only 31% classified with SUD by expert opinion was identified in administrative data [79] Another Nordic study found nearly 50% underreporting of SUD in individuals with schizophrenia in the Danish Psychiatric Register [82]. However, a sensitivity analysis identifying SUD patients from hospital data only did not change results appreciably in the study by Hjorthøj et al [30]. We had no information concerning frequency and level of past and current substance abuse, nor information concerning prescriptions for treatment of SUD. Finally, this study included only patients in specialized health care, possibly implying higher mortality than would be found among patients with SCZ and/or SUD in the general population. The generalizability of these findings is therefore to specialized care settings with similar health care systems and socioeconomic features.

## Conclusion

Our study demonstrates that patients with SCZ and/or SUD who receives specialized health care have a substantially elevated risk of premature mortality compared to the general population for both natural and unnatural causes of death. The high mortality in these vulnerable patients, and the differences in causes of death between the subgroups in our study, call for complex intervention strategies addressing several aspects of prevention, follow-up and treatment at all levels of primary and specialist health care. Young patients and women with SUD (with or without SCZ) have for unnatural causes particularly high mortality compared to the general population. The high proportion of deaths directly linked to poisoning in SUD patients urgently calls for a more effective societal prevention of SUD-related unnatural deaths.

### Disclaimer

Data from the Norwegian Patient Registry have been used in this publication. The interpretation and reporting of these data are the sole responsibility of the authors, and no endorsement by the Norwegian Patient Registry is intended nor should be inferred.

## Supporting information

S1 Table List of somatic diagnosis indicating alcohol abuse or drug abuse. (DOCX)

S2 Table Sensitivity-analysis for all-cause age, gender, and calendar-year standardized mortality ratios among men and women aged 20–79 with schizophrenia-related disorders (SCZ) and/or substance use disorders (SUD). (DOCX)

S3 Table All-cause age, gender, and calendar-year standardized mortality ratios among patients aged 20–79 with schizophrenia-related disorders (SCZ) and a concurrent substance use disorder (SUD), according to the temporal ordering of SCZ and SUD diagnosis. (DOCX)
