# Supplementary material for: Total and cause-specific standardized mortality ratios in patients with schizophrenia and/or substance use disorder
Source: PLoS One. 2018 Aug 23;13(8):e0202028. doi: 10.1371/journal.pone.0202028 (PMC6107156; doi:10.1371/journal.pone.0202028)
Supplement: S1 Table — (DOCX) [file pone.0202028.s001.docx]

**S1 Table. List of somatic diagnosis indicating alcohol abuse or drug abuse.**

| \| **ICD-10 code** \| **Description** \| \| --- \| --- \| \| E24.4 \| Alcohol-induced pseudo-Cushing syndrome \| \| E52 \| Niacin deficiency [pellagra] \| \| G31.2 \| Degeneration of nervous system due to alcohol \| \| G62.1 \| Alcoholic polyneuropathy \| \| G72.1 \| Alcoholic myopathy \| \| I42.6 \| Alcoholic cardiomyopathy \| \| K29.2 \| Alcoholic gastritis \| \| K70 \| Alcoholic liver disease \| \| K86.0 \| Alcohol-induced chronic pancreatitis \| \| O35.4 \| Maternal care for (suspected) damage to fetus from alcohol \| \| O35.5 \| Maternal care for (suspected) damage to fetus by drugs \| \| Z50.2 \| Alcohol rehabilitation \| \| Z50.3 \| Drug rehabilitation \| \| Z71.4 \| Alcohol abuse counselling and surveillance \| \| Z71.5 \| Drug abuse counselling and surveillance \| \| Z72.1 \| Alcohol use \| \| Z72.2 \| Drug use \| |
| --- | --- | --- | --- | --- | --- | --- | --- | --- | --- | --- | --- | --- | --- | --- | --- | --- | --- | --- | --- | --- | --- | --- | --- | --- | --- | --- | --- | --- | --- | --- | --- | --- | --- | --- | --- | --- |
