# Supplementary material for: Total and cause-specific standardized mortality ratios in patients with schizophrenia and/or substance use disorder
Source: PLoS One. 2018 Aug 23;13(8):e0202028. doi: 10.1371/journal.pone.0202028 (PMC6107156; doi:10.1371/journal.pone.0202028)
Supplement: S2 Table — (DOCX) [file pone.0202028.s002.docx]

**S2 Table. Sensitivity-analysis for all-cause age, gender, and calendar-year standardized mortality ratios among men and women aged 20-79 with schizophrenia-related disorders (SCZ) and/or substance use disorders (SUD).**

|  |  |  |  | SCZ-only | | |  | SUD-only | | |  | SCZ+SUD | | |
| --- | --- | --- | --- | --- | --- | --- | --- | --- | --- | --- | --- | --- | --- | --- |
|  |  |  |  | Obs | SMR | (95 % CI) |  | Obs | SMR | (95 % CI) |  | Obs | SMR | (95 % CI) |
| *Men* | |  |  |  |  |  |  |  |  |  |  |  |  |  |
|  | Original model | | | 972 | 4.5 | (4.2-4.8) |  | 7,334 | 6.4 | (6.2-6.5) |  | 423 | 7.6 | (6.9-8.3) |
|  | (i) | Excluding patients in somatic hospitals | | 678 | 3.8 | (3.5-4.1) |  | 3,419 | 5.7 | (5.5-5.9) |  | 324 | 7.8 | (7.0-8.7) |
|  | (ii) | Primary diagnosis only ^a^ | | 669 | 3.8 | (3.5-4.1) |  | 7,412 | 6.4 | (6.3-6.6) |  | 346 | 7.3 | (6.6-8.2) |
|  | (iii) | SCZ defined as F20 only | | 670 | 5.2 | (4.8-5.6) |  | 7,530 | 6.4 | (6.3-6.6) |  | 227 | 7.9 | (7.0-9.0) |
|  |  |  | |  |  |  |  |  |  |  |  |  |  |  |
| *Women* | | | |  |  |  |  |  |  |  |  |  |  |  |
|  | Original model | | | 832 | 4.3 | (4.0-4.6) |  | 2,592 | 7.4 | (7.1-7.7) |  | 165 | 7.0 | (6.0-8.2) |
|  | (i) | Excluding patients in somatic hospitals | | 591 | 3.6 | (3.3-4.0) |  | 1,226 | 6.2 | (5.9-6.5) |  | 105 | 6.5 | (5.4-7.9) |
|  | (ii) | Primary diagnosis only ^a^ | | 570 | 3.6 | (3.3-3.9) |  | 2,620 | 7.4 | (7.1-7.7) |  | 137 | 6.6 | (5.6-7.9) |
|  | (iii) | SCZ defined as F20 only | | 469 | 5.0 | (4.6-5.5) |  | 2,680 | 7.3 | (7.0-7.6) |  | 77 | 9.3 | (7.4-11.6) |
|  |  |  | |  |  |  |  |  |  |  |  |  |  |  |
| Abbreviations: Obs, observed deaths; SMR, standardized mortality ratio; 95 % CI, 95 % confidence interval; AUD, Alcohol Use Disorder. | | | | | | | | | | | | | | |
| ^a^ The number of patient in the SUD-only group increases, and the number of comorbid patients decreases, when a secondary diagnosis no longer qualifies for a SCZ diagnosis. | | | | | | | | | | | | | | |
