# Supplementary material for: Total and cause-specific standardized mortality ratios in patients with schizophrenia and/or substance use disorder
Source: PLoS One. 2018 Aug 23;13(8):e0202028. doi: 10.1371/journal.pone.0202028 (PMC6107156; doi:10.1371/journal.pone.0202028)
Supplement: S3 Table — (DOCX) [file pone.0202028.s003.docx]

**S3 Table. All-cause age, gender, and calendar-year standardized mortality ratios among patients aged 20-79 with schizophrenia-related disorders (SCZ) and a concurrent substance use disorder (SUD), according to the temporal ordering of SCZ and SUD diagnosis**

|  | 1 month | | |  | 3 months | | |  | 6 months | | |
| --- | --- | --- | --- | --- | --- | --- | --- | --- | --- | --- | --- |
|  | Obs | SMR | (95 % CI) |  | Obs | SMR | (95 % CI) |  | Obs | SMR | (95 % CI) |
| SCZ preceding SUD diagnosis | 189 | 5.5 | (4.8-6.4) |  | 161 | 5.2 | (4.4-6.1) |  | 146 | 5.3 | (4.5-6.2) |
| SUD preceding SCZ diagnosis | 167 | 8.4 | (7.2-9.8) |  | 145 | 7.9 | (6.7-9.3) |  | 121 | 7.6 | (6.4-9.1) |
| Both diagnosis within specified time frame | 217 | 9.8 | (8.6-11.2) |  | 267 | 9.9 | (8.8-11.1) |  | 306 | 8.3 | (8.3-10.4) |
| Abbreviations: Obs, observed deaths; SMR, standardized mortality ratio; 95% CI, 95% confidence interval | | | | | | | | | |  |  |
